# Xanthohumol: Anti-Inflammatory Effects in Mechanically Stimulated Periodontal Ligament Stem Cells

**DOI:** 10.3390/biomedicines12122688

**Published:** 2024-11-25

**Authors:** Christian Niederau, René H. Tolba, Joachim Jankowski, Nikolaus Marx, Michael Wolf, Rogerio Bastos Craveiro

**Affiliations:** 1 Department of Orthodontics, Medical Faculty, RWTH-Aachen University, 52074 Aachen, Germanyrcraveiro@ukaachen.de (R.B.C.); 2 Institute for Laboratory Animal Science and Experimental Surgery, Medical Faculty, RWTH-Aachen University, 52074 Aachen, Germany; 3 Institute of Molecular Cardiovascular Research, Medical Faculty, RWTH Aachen University, 52074 Aachen, Germany; 4 Experimental Vascular Pathology, Cardiovascular Research Institute Maastricht (CARIM), University of Maastricht, 6211 LK Maastricht, The Netherlands; 5 Aachen-Maastricht Institute for CardioRenal Disease (AMICARE), Medical Faculty, RWTH-Aachen University, 52074 Aachen, Germany; 6 Department of Internal Medicine I, Medical Faculty, RWTH-Aachen University, 52074 Aachen, Germany

**Keywords:** orthodontic tooth movement, human periodontal ligament stem cells (hPDLSCs), inflammation, cytokines, xanthohumol

## Abstract

Background/Objectives: Initial sterile inflammation is an essential molecular process in the periodontium during orthodontic tooth movement. A better understanding and possible modulations of these processes are of great interest to develop individual therapies for special patient groups. The prenylated plant polyphenol xanthohumol (XN) could have modulating effects as it has shown anti-inflammatory and angiogenesis-inhibiting effects in various cell lines. This study investigated the anti-inflammatory properties of XN in an in vitro model of compressively stimulated human periodontal ligament stem cells (hPDLSCs), which have a different function in the periodontium than the previously used cementoblasts. Methods: The expression of inflammatory markers at the mRNA and protein levels and the regulation of central kinases were investigated. Results: XN showed a dose-dependent influence on cell viability. Low concentrations between 0.2 and 4 µM showed positive effects, while 8 µM caused a significant decrease in viability after 24 h. Mechanical stimulation induced an upregulation of pro-inflammatory gene (IL-6, COX2) and protein (IL-6) expression. Here, XN significantly reduced stimulation-related IL-6 mRNA and gene expression. Furthermore, the phosphorylation of AKT and ERK was upregulated by mechanical stimulation, and XN re-established phosphorylation at a level similar to the control. Conclusions: We demonstrated a selective anti-inflammatory effect of XN in hPDLSCs. These findings provide the basis for further investigation of XN in the modulation of inflammatory responses in orthodontic therapy and the treatment of periodontal inflammation.

## 1. Introduction

Xanthohumol (XN), a prenylated flavonoid found in hops, exhibits various biological activities. It possesses potent anti-inflammatory, antioxidant, and anti-cancer properties [1,2,3]. XN has exhibited anti-inflammatory activity by suppressing the production of pro-inflammatory cytokines and enzymes, attenuating oxidative stress, and modulating the MAPK and PKB pathways [3,4,5,6]. The molecular target of XN remains unclear. Furthermore, dose-dependent viability-enhancing effects are known [7]. Given the documented anti-inflammatory properties of XN, exploring its potential effects on sterile inflammation during orthodontic tooth movement could be of significant interest, with the aim of developing novel therapeutic strategies to prevent side effects of orthodontic therapy.

Orthodontic tooth movement, a widely employed dental treatment modality, involves applying controlled forces to induce tooth displacement within the periodontal ligament to improve functional and esthetical properties of the dentition. The periodontal ligament is a specialized connective tissue situated between the tooth and the alveolar bone, consisting of collagen fibers, fibroblasts, blood vessels, and nerves. The principal function is to anchor the tooth to the jaw. This is achieved through collagen fibers synthesized by periodontal ligament stem cells (hPDLSCs) exhibiting a fibroblastic phenotype. These fibers are embedded in bone and in cementum on the tooth root surface [8,9,10].

When orthodontic forces are applied, the teeth move slightly within the alveolar socket, and thus, areas of compression or tension occur in the periodontal ligament [11]. This mechanical stimulation induces an intricate interplay of cellular and molecular events within the periodontal ligament, including the release of cytokines, chemokines, and growth factors, as well as the activation of inflammatory pathways [12,13]. These processes are tightly regulated and contribute to the recruitment and activation of various cell types, such as osteoclasts and osteoblasts, which remodel the alveolar bone and periodontal ligament, enabling tooth movement by resorbing or producing alveolar bone. Attenuation of the inflammatory process results in less orthodontic tooth movement, while pro-inflammatory drugs increase it [14,15,16,17]. However, dysregulated or excessive inflammation can lead to unintended tissue damage in the form of irreversible tooth root resorptions [18,19]. The risk is anticipated to be elevated in patients with concurrent pathogen-associated inflammatory conditions, such as periodontitis.

At this point, the modulation of inflammatory processes inside the periodontal ligament with anti-inflammatory agents such as XN is of high interest. A comprehensive understanding of these processes’ regulatory mechanisms is essential to mitigate orthodontic side effects, such as severe root resorption in compressed periodontal ligament areas, which may be associated with dysregulation at the periodontal ligament–cementum interface. Therefore, the modulation of XN has potential benefits not only in orthodontic therapy but also for the pathogenesis of periodontal tissues, such as gingivitis or periodontitis [20].

The biochemical response to mechanical stimulation is known to be dominated by hPDLSCs, activating the regulation of bone remodeling and tissue regeneration [21]. Inflammatory signaling cascades play a critical role in this process through the release of various pro-inflammatory cytokines and cell death mediators. Interleukin-6 and -8 (IL-6 and IL-8), as well as cyclooxygenase-2 (COX-2), tumor necrosis factor-α (TNF-α), and toll-like receptor (TLR) ligands have been shown to play an essential role [22,23,24,25]. These pro-inflammatory cytokines and cell death mediators play a critical role in the early phase of sterile inflammation during orthodontic tooth movement and other inflammatory responses, such as gingivitis and periodontitis [26]. However, the function of hPDLSCs during orthodontic tooth movement is not fully understood. Therefore, further studies are needed to investigate the processes inside the periodontal ligament involved in adapting and remodeling during mechanical loading.

This study aims to investigate the potential of XN to modulate the non-pathogen-associated inflammatory response of hPDLSCs to mechanical stimulation, utilizing a well-established in vitro model of orthodontic tooth movement.

## 2. Materials and Methods

### 2.1. Reagents and Antibodies

Xanthohumol was purchased from Carl Roth (Karlsruhe, Germany), solved in Dimethylsulfoxide (DMSO) (Carl Roth, Karlsruhe, Germany), and stored at −20 °C in 10 mM aliquots. The aliquots were used a single time to avoid freeze/thaw degradation cycles. The primary Western blot antibodies Phospho-Akt (Ser473) (D9E), dilution 1:2000 (#4060); Phospho-p44/42 MAPK (Erk1/2) (Thr202/Tyr204) (D13.14.4E), dilution 1:2000 (#4370); AKT (40D4), dilution 1:2000 (#2920); p44/42 MAPK (Erk1/2) (3A7), dilution 1:1000 (#9107); and GAPDH (14C10), dilution 1:1000 (#2118S) were purchased from CellSignaling, Danvers, MA, USA. The secondary antibodies StarBrightBlue 700 (#12004161) and StarBrightBlue 520 (#12005866) were purchased from BioRad, Hercules, CA, USA.

### 2.2. Cell Culture and Mechanical Stimulation

PDLSCs were obtained from extracted healthy human teeth from the upper jaw of three different donors according to our previous study [27]. The same donors were used in this study as before. The collection and usage of hPDLSCs from discarded patient biomaterial were approved by the ethics committee of the University Clinics of RWTH Aachen, Germany (approval number EK 374/19), and all experiments were carried out in accordance with the relevant guidelines and regulations. The cells were cultured in DMEM high glucose (1 g/L) (Gibco, Waltham, MA, USA), 10% FCS (Gibco, Waltham, MA, USA), 50 mg/L ascorbic acid (Sigma, Saint Louis, MO, USA), 100 units/mL of penicillin, and 100 μg/mL of streptomycin (Gibco, Waltham, MA, USA) in cell culture plates at 37 °C and 5% CO_2_ in a humidified atmosphere. Cells were trypsinized, centrifuged at 350× *g*, and quantified using a Neubauer Counting Chamber, and 80,000 cells were plated in each 6-well plate for quantitative real-time-RT-PCR analysis and Western blotting. Upon reaching a confluence of 90% after 48 h, cells were mechanically stimulated with glass cylinders, which were placed on the cell monolayer, exerting a static force (2 g/cm^2^) for 6 h and 24 h. This compression method has already been established for hPDLSCs and described in previous publications [28,29,30,31,32]. All experiments were performed with isolated cells from the three different donors cultured with DMEM high glucose and 1% FCS. The use of cells from three different donors was intended to avoid bias in the results due to individual peculiarities, as can occur with primary cells.

### 2.3. MTS Assay

An MTS assay was performed in 96-well plates in 50 µL 1% FCS medium with 3000 cells/well. XN was added in concentrations ranging from 0.2 µM up to 8 µM after 24 h of acclimatization by adding 50 µL of medium with double the XN concentration needed to obtain the correct concentrations in the wells. After 6, 12, 24, and 48 h, 20 µL of CellTiter 96^®^ AQueous One Solution Cell Proliferation Assay (MTS) (Promega, Madison, WI, USA) was added. Absorption was measured using an ELISA plate reader (Tecan, Männedorf, Switzerland) after 4 h of incubation according to the manufacturer’s protocol. Cell viability was calculated relative to the control, represented by cells treated with DMSO without XN.

### 2.4. Isolation and Purification of RNA

For RNA isolation, cells in each well were first washed with 2 mL phosphate-buffered saline (Gibco) and then harvested with 0.5 mL TRIzol^TM^ Reagent (Thermo Fisher Scientific, Waltham, MA, USA). The RNA yield of each sample was checked photometrically at 280 nm and 260 nm (Nanodrop OneTM, Thermo Fisher Scientific, Waltham, MA, USA) after isolation according to the manufacturer’s instructions. RNA was then purified using the RNeasy Mini Kit (Qiagen, Hilden, Germany) according to the manufacturer’s protocol, including on-column DNA digestion (RNase-Free DNase, Qiagen, Hilden, Germany). In order to ascertain the efficacy of the purification process and to obtain data regarding the yield of RNA for the purpose of ensuring uniform cDNA synthesis, each sample was subjected to a second photometric measurement following the completion of the purification procedure.

### 2.5. RT-qPCR

The RNA was transcribed into cDNA (SuperScript III RT, Thermo Fisher Scientific, USA). Using the measurement after RNA purification, the final cDNA concentration was adjusted to 25 ng/μL. All steps from RNA isolation to cDNA synthesis were performed in parallel for all samples of each experiment to avoid experimental variations. The differences in mRNA gene expression were determined by a real-time quantitative polymerase chain reaction (RT-qPCR) in technical duplicates using 2.5 ng/μL cDNA in each reaction and a primer concentration of 0.5 μM. Primers (Table 1) were self-designed as previously described [7,30]. All RT-qPCR data were normalized by the delta–delta Ct method to the reference gene *Rpl22* and to the unstimulated control.

### 2.6. Western Blot

For the extraction of cellular proteins, hPDLSCs were lysed on ice with Pierce RIPA buffer (Thermo Fisher Scientific, USA, #89900) with added cOmplete Mini Tablets (Roche Holding, CHE, #04693124001) and PhosStop (Roche Holding, CHE, #04906837001). Subsequently, lysate was centrifuged (10 min, 20,000× *g*, 4 °C). The protein concentration of cell lysates was analyzed using a Bradford assay (Bio-Rad, Hercules, CA, USA). Proteins (15 µg) were loaded onto 12% SDS-polyacrylamide gels (#1610185, Bio-Rad, Hercules, CA, USA) and transferred onto nitrocellulose membranes in an amount of 0.2 µm (#1704270, Bio-Rad, Hercules, CA, USA). After being blocked in 5% BSA in TBS-T (TRIS-buffered saline and 0.1% Tween-20) for one hour at RT, membranes were incubated at 4 °C overnight with primary antibodies. The immunoreactive bands were detected by using fluorescent secondary antibodies with the ChemiDoc MP imaging system (Bio-Rad, Hercules, CA, USA).

### 2.7. ELISA

To examine the translational level, an enzyme-linked immunosorbent assay (ELISA) kit for IL-6 (CSB-E04638h, Cusabio Wuhan Huamei Biotech Co., Wuhan, China) was used using fresh cell culture supernatant, following the instructions provided by the manufacturer.

### 2.8. Statistical Analysis

Normal distribution was tested using the Shapiro–Wilk test, and homogeneity of variance using the Brown–Forsythe test, followed by ANOVA analysis of variance with Tukey’s post hoc test (Prism version 10.2.3; GraphPad Software), where *p* < 0.05 was considered statistically significant. Graphs show mean values ± standard deviation (SD).

## 3. Results

### 3.1. Compressive Force Led to Decreased Growth Density

**Figure 1:**
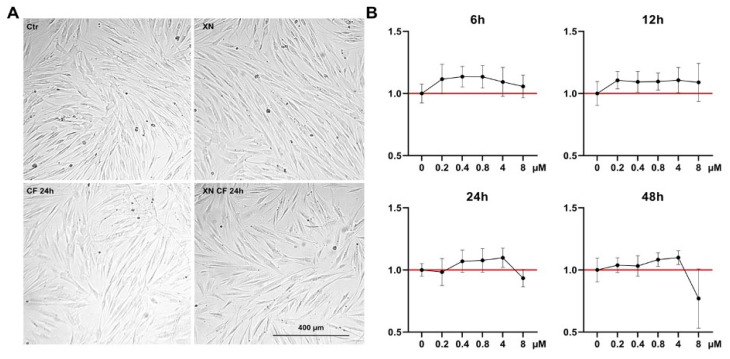
Cell morphology and viability. (**A**) Morphology of hPDLSCs under mechanical stimulation (CF) with and without Xanthohumol (XN). Twenty-four-hour Compressive force led to slightly reduced growth density compared to control. Addition of XN had no impact on proliferation and cell morphology. (**B**) MTS assay with xanthohumol concentrations from 0 up to 8 µM, analyzed 6, 12, 24, and 48 h after application. Data from cells from three different donors are shown. Low doses enhanced cell viability, while 4 and 8 µM exerted cytotoxic effects, especially at late time points. Normalized to control (red line).

Static mechanical stimulation with a compressive force of 2 g/cm^2^ resulted in reduced cell confluence compared to the unstimulated control condition. The addition of XN had no effect on cell proliferation. Cell morphology was observed by light microscopy and was not affected by XN (Figure 1A).

### 3.2. The Viability of hPDLSCs Was Increased and Decreased by Xanthohumol in a Time- and Dose-Dependent Manner

Even low concentrations of 0.2 µM showed a viability-promoting effect. This effect was stronger at short (6 h and 12 h) than at longer durations (24 h and 48 h). At 6 h and 12 h, increasing the concentration from 0.2 to 8 µM led to an increase in the effect. After 24 h and 48 h, in contrast, a stronger effect was observed at higher doses of up to 4 µM. However, a clear deterioration in viability was observed at these time points due to the 8 µM concentration. Such effects were not evident after 6 h and 12 h. Overall, viability-promoting effects of XN were observed at short time points, whereas viability-inhibiting effects were increased at longer time points (Figure 1B).

### 3.3. XN Reduces IL-6 mRNA Expression in Compressively Stimulated hPDLSCs

**Figure 2:**
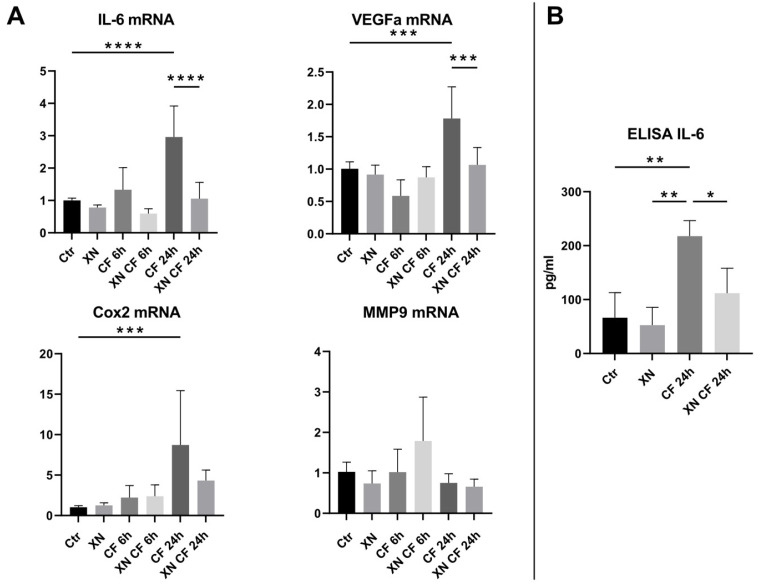
RT-qPCR and ELISA of pro-inflammatory markers. Effect of xanthohumol (XN) on hPDLSCs stimulated with static compressive force (CF). (**A**) Gene expression results of inflammatory markers. Data were normalized to the reference gene *RPL22*, expressed as fold of control, which was set to 1. Values represent mean ± SD of two independent experiments. (**B**) Regulation of IL-6 quantified by ELISA. * *p* < 0.05 was considered statistically significant by ANOVA followed by Tukey’s multiple comparisons test. (** *p* < 0.01, *** *p* < 0.001, **** *p* < 0.0001).

We investigated how XN can influence the already known increase in the expression of inflammatory markers through the mechanical stimulation of hPDLSCs. Therefore, we analyzed the pro-inflammatory genes *IL-6*, *VEGFA*, *COX2*, and *MMP9* expression. We found a significant increase in *IL-6* (*p* < 0.0001), *VEGFA* (*p* = 0.0003), and *COX2* (*p* = 0.001) after 24 h under compressive force. In contrast, short stimulation of 6 h did not show any significant effect. XN did not affect the basal gene expression of any of the investigated markers. In stimulated conditions (24 h), XN significantly prevented the increase in the mRNA expression of *IL-6* (*p* < 0.0001) and *VEGFA* (*p* = 0.0009) which stayed on the basal level. A comparable profile was detectable for *COX2* without reaching a level of significance. In contrast, mechanical stimulation (6 h and 24 h) and XN did not affect *MMP9* mRNA expression (Figure 2A).

### 3.4. Xanthohumol Increased the Levels of IL-6 Protein Expression from Compressively Stimulated hPDLSCs

After the addition of compressive strain, IL-6 was significantly (*p* = 0.0067) upregulated on the protein level. Furthermore, we confirmed that IL-6 protein expression was reduced (*p* = 0.0443) by the addition of 0.8 µM XN under compressive force. However, IL-6 expression was still increased compared to the controls. XN did not affect basal IL-6 expression (Figure 2B).

### 3.5. The Phosphorylation of AKT and ERK Induced by Compressive Stimulation Was Re-Established by Xanthohumol

**Figure 3:**
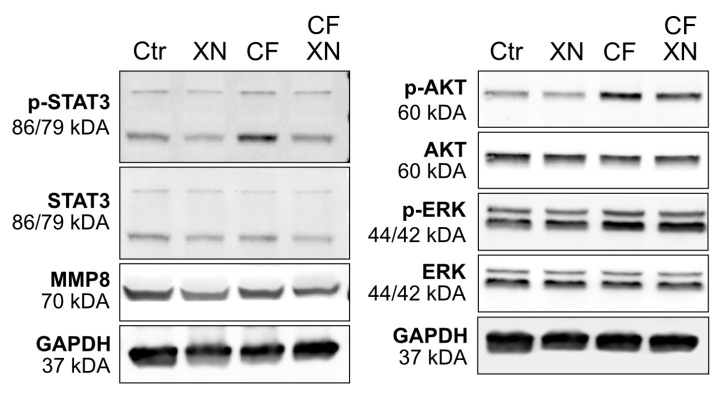
Effects of xanthohumol (XN) on expression and phosphorylation of MAP-kinase ERK, protein kinase b (AKT), Signal transducer and activator of transcription 3 (STAT 3), and matrix metalloproteinase-8 (MMP8) in mechanically stimulated hPDLSCs. GAPDH served as loading control. Representative blots of three independent experiments are presented. Phosphorylated variants are labeled as “p-”. CF = stimulation with static compressive force.

Through a Western blot analysis, we investigated pathways involved in hPDLSC cytokine expression, such as MAP-kinase ERK, protein kinase B (AKT), and STAT3 and their activated/phosphorylated form. The phosphorylation of ERK, AKT, and STAT3 was upregulated in probes with mechanical strain after 24 h. The addition of XN decreased this upregulation without reaching the level of basal conditions. An effect on the phosphorylation of AKT in unstimulated probes was not detectable. Interestingly, XN slightly reduced the phosphorylation of ERK and STAT3 in these conditions. MMP8 was not affected by mechanical stimulation. However, minor expression-increasing XN effects were detectable in basal and unstimulated conditions (Figure 3).

## 4. Discussion

This study focused on examining sterile inflammatory processes in hPDLSCs under mechanical stimulation, simulating conditions associated with orthodontic tooth movement. These molecular processes enable targeted remodeling of the alveolar bone and the periodontal ligament, allowing the movement of a tooth through the bone. In rare cases, excessive reactions of this system lead to undesirable side effects in the form of root resorption and excessive vertical alveolar bone loss. Therefore, the possibility of modulating these processes is of great clinical interest. The active herbal ingredient xanthohumol has already shown various anti-inflammatory, anti-angiogenic, and anti-proliferative effects in numerous studies that have focused mostly on cancer research [2,33,34,35]. In our previous publication, we demonstrated anti-inflammatory and cell viability-promoting effects of xanthohumol in immortalized murine dental cementoblasts [7]. However, the current manuscript takes this a step further with another cell type of the periodontium, which has a different function to the cementoblasts that we have previously published. We use self-isolated primary periodontal stem cells derived from human donor teeth, which have been extensively characterized in a previous publication. Therefore, our results show more reliable data obtained with human cell material that was not immortalized. We confirmed the anti-inflammatory properties of xanthohumol on primary human periodontal ligament stem cells.

Low concentrations of xanthohumol were used, which are comparable to the doses that a person may come into contact with in everyday life when consuming normal beer (0.4 µM) [36] or beer enriched with XN (8.5 µM) [37]. Moreover, these concentrations were analogous to the total XN concentrations (free and conjugated forms) observed in human blood plasma following oral XN administration [38]. The ratio of conjugated XN to free XN in human plasma is nearly 100% in favor of the conjugated form [38]. In numerous instances, conjugated agents do not contribute significantly to therapeutic effects when compared to their free counterparts. However, in the context of XN, biological effects appear to correlate with total XN levels (conjugated and free), rather than solely with levels of free XN [38,39,40]. Additionally, XN has demonstrated high bioavailability and a low toxicity profile [41,42]. It has been established that orally administered xanthohumol does not adversely impact major organ functions in vivo [43].

The in vitro experiments in this study revealed effects of XN on cytotoxicity and cell viability depending on the used concentration. Low doses showed viability-promoting effects, while higher doses had cytotoxic effects. This profile was comparable to our previous experiments with cementoblasts [7]; however, the hPDLSCs appeared to be more resistant against cytotoxic effects. Based on the viability assay, an XN concentration of 0.8 µM was used for further experiments with compressive stimulation. The amount of force exerted was comparable to previous experiments with periodontal fibroblasts [27,44,45] and cementoblasts [7,29,30].

Mechanical stimulation led to the upregulation of pro-inflammatory markers such as *IL-6* and *COX2* on an mRNA level. These findings are in line with previous publications [27,46]. Following our hypothesis, XN decreased compression-dependent IL-6 upregulation on the mRNA and protein levels. IL-6 is a well-known marker of periodontal remodeling, involved in osteoclast differentiation and activation [25]. The capacity of XN to affect IL-6 expression suggests that XN is a possible agent for modulating inflammatory processes inside the periodontal ligament.

XN is also known to have anti-angiogenic properties. We therefore investigated the regulation of *VEGFA*, which is upregulated by mechanical stimulation in periodontal ligament cells and cementoblasts [7,22]. In contrast to our experiments with cementoblasts, XN was able to reduce the mRNA upregulation of *VEGFA* to a basal level.

Previous studies with various cancer cell lines have demonstrated that XN interacts with MAP-kinases ERK [4,47,48], JNK [4], and p38 [5] and with protein kinase B/AKT [3,6,49]. These kinases are well-known for their involvement in cell proliferation, cell survival, cell stress, and inflammatory responses. We and other groups have already reported that phospho-ERK and phospho-AKT are upregulated by mechanical stimulation in periodontal cells [27,29,50]. This study demonstrated that XN reduced the levels of both phosphorylated forms. ERK, activated by phosphorylation, can translocate into the nucleus and modulate genes to respond to cellular stimulation [51,52]. Hence, a correlation of ERK activation and IL-6 expression caused by mechanical stimulation in periodontal ligament cells can be hypothesized.

Signal transducer and activator of transcription (STAT) proteins play a pivotal role in the production of inflammatory cytokines. Among these proteins, STAT3 is particularly crucial for regulating the expression of cytokines, chemokines, and other mediators that can induce and perpetuate an inflammatory environment [53]. This study provides the first data indicating that STAT3 is activated in conditions with mechanical stimulation in periodontal cells. Furthermore, XN was able to reduce this activation. This makes XN interesting for modulating inflammatory processes inside the periodontal ligament.

The results of this study must be seen considering certain limitations, since we could not describe or suggest a cell signaling mechanism for XN. Furthermore, the presented data are based on in vitro experiments with isolated human cells from three different donors. Further investigations are needed to prove the potential of XN as an anti-inflammatory agent in human therapy.

## 5. Conclusions

In the present study, we demonstrated the selective anti-inflammatory effects of xanthohumol in human primary periodontal ligament stem cells. These findings were obtained using primary human periodontal ligament stem cells, which have a different function in the periodontium than the previously used immortalized murine cementoblasts. These results may represent a promising approach for modulating inflammatory responses within the periodontal ligament, particularly in orthodontic therapy and treatment for periodontitis. Our findings may contribute to the development of new therapeutic strategies to increase the effectiveness of orthodontic treatment and improve the comfort of patients undergoing therapies with orthodontic tooth movement.

## Figures and Tables

**Table 1:** RT-qPCR gene, primer and target/amplicon information for the reference gene *RPL22* and investigated target genes. Tm: melting temperature of primer/specific qPCR product (amplicon); %GC: guanine/cytosine content; bp: base pairs.

Gene Symbol	Gene Name (Homo Sapiens)	Gene Function	Accession Number (NCBI Gene Bank)	Chromosome Location (Length)	5′ Forward Primer-3′ (Length/Tm/GC)	5′ Reverse Primer-3′ (Length/Tm/GC)	Primer Location	Amplicon Length	Amplicon Location (bp of Start/Stop)	Intron-Flanking (Length)	Variants Targeted (Transcript/Splice)
*RPL22*	ribosomal protein L22	translation of mRNA in protein	NM_000983	1; 1p36.31	TGATTGCACCCACCCTGTAG	GGTTCCCAGCTTTTCCGTTC	Exon 2/3	98	91/188	yes	yes
				(2061 bp)	(20 bp/59.67 °C/55%GC)	(20 bp/59.4 °C/55%GC)					
*IL-6*	Interleukin 6	important role in bone metabolism; osteoclastogenesis	NM_000600	7; 7p15.3	CATCCTCGACGGCATCTCAG	TCACCAGGCAAGTCTCCTCA	Exon 2/4	164	240/403	yes	yes
				(1127 bp)	(20 bp/60.32 °C/60%GC)	(20 bp/60.47 °C/55%GC)					
*MMP9*	matrix metalloprotease 9	breakdown of extracellular matrix; reproduction and tissue remodelling	NM_004994.3	20q13.12	ATTTCTGCCAGGACCGCTTC	TCATAGGTCACGTAGCCCACT	Exon 13	85	2053/2118	no	no
				(2336 bp)	(20 bp/60.68 °C/55%GC)	(21 bp/60.34 °C/52,38%GC)					
*VEGFA*	vascular endothelial growth factor A	induces proliferation and migration of vascular endothelial cells	NM_001171623	6p21.1	GGAGGGCAGAATCATCACGAA	GGTACTCCTGGAAGATGTCCAC	Exon 2/3	100	1153/1211	yes	yes
				(3660 bp)	(21 bp/60.1 °C/52.3%GC)	(22 bp/59.8 °C/54.5%GC)					
*PTGS2 (COX2)*	prostaglandin-endoperoxide synthase 2	involved in prostaglandin synthesis	NM_000963	1q31.1	GATGATTGCCCGACTCCCTT	GGCCCTCGCTTATGATCTGT	Exon 4/5	185	560/725	yes	yes
				(4510 pb)	(20 bp/59.8 °C/55%GC)	(20 pb/59.6 °C/55%GC)					

## Data Availability

The data that support the findings of this study are available from the corresponding author upon reasonable request.
